# Perhaps fewer, but faster: should we rethink the 90% stroke unit admission standard?

**DOI:** 10.1093/esj/aakag057

**Published:** 2026-06-08

**Authors:** Umberto Pensato, Aravind Ganesh, Nishita Singh, Simona Marcheselli, Andrew M Demchuk

**Affiliations:** Department of Biomedical Sciences, Humanitas University, Milan, Italy; IRCCS Humanitas Research Hospital, Milan, Italy; Department of Clinical Neurosciences, University of Calgary, Calgary, AB, Canada; Department of Internal Medicine (Neurology Division), Rady Faculty of Health Sciences, University of Manitoba, MB, Winnipeg, Canada; IRCCS Humanitas Research Hospital, Milan, Italy; Department of Clinical Neurosciences and Radiology, Hotchkiss Brain Institute, Cumming School of Medicine, University of Calgary, Calgary, Canada

**Keywords:** acute treatment, cerebrovascular disease, myocardial infarction, prognosis, stroke care

Stroke remains among the most burdensome conditions in Europe, and its societal and individual impact continues to grow in the context of an ageing population. This underscores the importance of the mid-term review and update of the Stroke Action Plan for Europe 2018–2030 (SAP-E),^1^ a comprehensive framework encompassing the entire stroke care continuum and defining measurable, accountable targets for 2030. The incorporation of Key Performance Indicators alongside the Stroke Service Tracker within a Europe-wide governance structure represents an important advancement, enabling standardised monitoring, enhanced accountability and more effective evaluation of progress in stroke care delivery.

However, we wish to raise concerns regarding the recommendation that >90% of patients with acute stroke be admitted to a stroke unit within 24-h as first level of care. While we fully endorse the pivotal role of stroke units for most patients, we believe this universal target warrants critical reappraisal in light of clinical, demographic, ethical and operational realities.

## Stroke Unit evidence does not cover all patient populations

Stroke Unit care is among the most robustly evidence-based interventions in medicine.^2^ However, in landmark trials comparing stroke units with general wards, patients with severe pre-existing disability, advanced dementia and extreme frailty were underrepresented. In these populations, potential benefits appear largely driven by reduced mortality rather than meaningful functional recovery.^2^

For patients experiencing stroke at the end-of-life, medical priorities should be guided by patient preferences and goals of care. Stroke units are designed for intensive monitoring, standardised pathways, aetiological workup and early secondary prevention. Yet for patients with a pre-existing mRS = 5, severe dementia, or terminal malignancy, identifying untreatable aetiology or monitoring for complications that cannot be addressed offers very limited benefit. In such cases, a palliative or comfort-focused approach may be more appropriate and aligned with patients wishes.^3^ Moreover, a stroke unit, with continuous monitoring, alarms and restricted visiting, is rarely the right environment for end-of-life care.

## Demographic pressures and sustainability

With life expectancy exceeding 80-year in many European countries, stroke increasingly affects the very old, the multimorbid and those with pre-existing disability. Applying a blanket 90% stroke unit admission target to this evolving population raises sustainability concerns. Stroke units depend on specialised staff and infrastructure that are inherently limited. Admitting patients unlikely to derive measurable benefit may strain resources and delay access for those who need them most. Timely stroke unit admission is particularly critical for patients at high risk of early neurological deterioration, where specialised neuroscience nursing expertise (including NIHSS certification) is crucial. When stroke units operate at full capacity such patients may remain in emergency department for prolonged periods, where the hectic environment may also impact adherence to key aspects of care (eg, achieving BP targets in ICH).

## The 24-h admission window

The SAP-E target specifies that 90% of patients should be admitted to a stroke unit within 24 h of hospital arrival.^1^ While pragmatic, the therapeutic value of stroke unit care is greatest in the earliest hours after onset, when reperfusion therapies are administered and neurological deterioration or arterial access site complications are most likely.^4^ In systems using a 24-h threshold, admission at hour 23 may be considered compliant despite missing this critical window. We suggest complementing this metric with a more stringent target, such as admission within 2–6 h for high-risk patients, particularly those receiving reperfusion therapies.

## A lesson from cardiology

Not all patients with myocardial infarction are admitted to coronary care units.^5^ Clinical triage—guided by age, functional status, comorbidity and likelihood of benefit from invasive monitoring and intervention—determines the appropriate level of care. Patients with limited life expectancy or comfort-oriented goals are appropriately managed in general or palliative settings, without this being considered a system failure. Conversely, high-risk patients are usually moved swiftly from intervention to dedicated coronary care units. Stroke care should evolve towards a similarly nuanced model.

## Conclusion

A target of 90% stroke unit admission is appropriate for patients for whom active treatment is indicated and potentially beneficial. However, for those at the end of life, with severe pre-existing disability or with clearly expressed preferences against intensive intervention, the same target risks promoting care that is neither appropriate nor aligned with patient values.

What is required is an early conversation with patients and families regarding goals of care before admission decisions. For some, this will support palliative approach outside stroke units.^3^ Such an approach would preserve specialised resources for patients most likely to benefit (eg, early admission for thrombectomy-treated patients), while ensuring that patients at the end of life receive dignified care concordant with their wishes.
